# Social and emotional processing in Prader-Willi syndrome: genetic subtype differences

**DOI:** 10.1186/1866-1955-5-7

**Published:** 2013-03-27

**Authors:** Alexandra P Key, Dorita Jones, Elisabeth M Dykens

**Affiliations:** 1 Vanderbilt Kennedy Center for Research on Human Development, Vanderbilt University, 230 Appleton Place, Peabody Box 74, Nashville, TN, 37203, USA; 2 Department of Hearing and Speech Sciences, Vanderbilt University Medical Center, 1215 21st Ave South, Rm. 8310, Nashville, TN, 37232, USA

**Keywords:** Prader-Willi syndrome, Face perception, Emotion processing, Attention, Event-related potential

## Abstract

**Background:**

People with Prader-Willi syndrome (PWS) demonstrate social dysfunction and increased risk of autism spectrum disorder, especially those with the maternal uniparental disomy (mUPD) versus paternal deletion genetic subtype. This study compared the neural processing of social (faces) and nonsocial stimuli, varying in emotional valence, across genetic subtypes in 24 adolescents and adults with PWS.

**Methods:**

Upright and inverted faces, and nonsocial objects with positive and negative emotional valence were presented to participants with PWS in an oddball paradigm with smiling faces serving as targets. Behavioral and event-related potential (ERP) data were recorded.

**Results:**

There were no genetic subtype group differences in accuracy, and all participants performed above chance level. ERP responses revealed genetic subtype differences in face versus object processing. In those with deletions, the face-specific posterior N170 response varied in size for face stimuli versus inverted faces versus nonsocial objects. Persons with mUPD generated N170 of smaller amplitude and showed no stimulus differentiation. Brain responses to emotional content did not vary by subtype. All participants elicited larger posterior and anterior late positive potential responses to positive objects than to negative objects. Emotion-related differences in response to faces were limited to inverted faces only in the form of larger anterior late positive potential amplitudes to negative emotions over the right hemisphere. Detection of the target smiling faces was evident in the increased amplitude of the frontal and central P3 responses but only for inverted smiling faces.

**Conclusion:**

Persons with the mUPD subtype of PWS may show atypical face versus object processes, yet both subtypes demonstrated potentially altered processing, attention to and/or recognition of faces and their expressions.

## Background

Prader-Willi syndrome (PWS) is a genetic disorder associated with a deletion on paternal chromosome 15q11-13 (deletion subtype, 70% of cases) or duplication of the maternal chromosome (maternal uniparental disomy (mUPD), 25% of cases) [1,2]. The phenotype includes intellectual disabilities, compulsivity, hyperphagia, and increased risks of life-threatening obesity [3,4]. Several studies have examined possible phenotypic differences in PWS across these two major genetic subtypes in neuroanatomy [5], cognitive performance and adaptive skills [6-8], food-related behaviors [9,10], and behavioral problems and psychiatric illness [11-15].

Recently, the PWS phenotype description has been expanded to include an increased risk of autism-spectrum symptomatology, especially in persons with the mUPD subtype [4,13,16]. Autism spectrum disorders (ASD) include a triad of impairments in social and communicative functioning as well as the presence of repetitive behaviors and interests [17]. Most individuals with PWS do not meet full criteria for a diagnosis of ASD [18], but compared with others with intellectual disabilities are more similar to those with ASD in their repetitive behaviors and social functioning [16]. Although several studies have compared phenotypic features of PWS versus ASD (for a review see [19]), studies of the social impairments that characterize PWS, including possible mechanisms associated with these difficulties, are just beginning [20].

In persons with ASD, symptom severity in the social domain often correlates with deficits in perceptual face processing [21,22]. While the range of performance on tasks involving faces is wide [23], deficits appear to be most pronounced in more demanding tasks, such as those involving emotional expressions [24] (for a review see [25]). Recently, García-Villamisar and colleagues demonstrated that emotion recognition abilities and not face perception *per se* are associated with social adaptive functioning in adults with ASD [26].

Individuals with PWS also appear to have difficulties processing facial emotional expressions. These difficulties are reflected in their poor performance on labeling complex emotional expressions (depicted by photographs of the eye region) [27] and limited emotion recognition beyond the extreme happy and sad expressions [28]. In a recent study comparing parental reports with their child’s actual ability to recognize emotional faces, Whittington and Holland observed that parents correctly judged the ability of their children with PWS to recognize happiness, yet overestimated their accuracy of recognizing sadness [15]. Neither the overall accuracy of participants nor their recognition of specific emotions was related to their genetic subtype, but correlated with their intellectual quotient (IQ) and socialization scores [15].

Behavioral assessments of face processing in individuals with developmental disabilities may be challenging due to the need for participants to comprehend instructions and provide overt responses. Psychophysiological measures, such as event-related potentials (ERPs), have minimal cognitive demands, as they do not require behavioral responses to document processing of presented information. ERPs can thus circumvent challenges in behavioral testing, while reflecting even subtle individual differences in performance. Previous ERP research on face processing in typical adults has identified a specific negative peak that is maximal over the occipito-temporal scalp regions at 170 ms after stimulus onset (N170), originates in the fusiform gyrus [29,30], and is sensitive to faces. This peak is significantly larger in response to faces than objects [31-34] and for inverted compared with upright faces [35,36]. In participants with ASD, the N170 response usually has a smaller than typical amplitude [37,38], atypical scalp distribution [37,39-41], delayed latency [40], and appears insensitive to face orientation [40,42]. In the only ERP study of face perception in PWS involving passive viewing of upright or inverted faces with direct or averted gaze, Halit and colleagues reported that while participants with both genetic subtypes generated delayed N170 responses to inverted faces regardless of gaze direction, in persons with mUPD the N170 amplitude varied based on face orientation (larger for inverted faces) and gaze direction (larger for averted gaze) [43]. These findings suggest that adults with the deletion subtype resembled individuals with ASD with regard to reduced sensitivity to face orientation, while brain responses of adults with the mUPD subtype were similar to those of individuals with ASD in relation to gaze direction.

Importantly, however, success in social interactions depends not only on the ability to process faces differently from objects but also on the more complex ability to understand the facial expressions of emotion, a skill that may be atypical both in ASD and PWS. Even so, electrophysiological responses associated with processing of emotional information in faces have not been extensively studied in individuals with developmental disabilities. One ERP study in children with ASD suggested reduced sensitivity to emotional expressions as reflected by the lack of modulation of N300 (precursor of the adult N170) response to fearful versus neutral faces [39], while others observed no emotion-related differences in ERPs of children or adults with autism or Asperger’s syndrome [38,44].

These conflicting findings regarding emotion processing in ASD could be explained by the specific ERP response chosen for analysis. In typical populations, some studies report modulation of N170 by emotional expression (for example, [45-47]) and others observe no effects [48,49]. However, a different ERP response – late positive potential (LPP) recorded over centro-parietal as well as frontal scalp regions – is known to vary between emotional and neutral stimuli [50,51]. This response begins 300 to 500 ms after stimulus onset regardless of whether participants are explicitly asked to evaluate emotional content [52]. LPP responses are not face specific, and have been recorded to a wide range of affective stimuli including pictures of faces, scenes, objects, and words, and in tasks that required explicit evaluation as well as passive viewing (for a review see [53]). Furthermore, while the centro-parietal LPP response may not distinguish between positive and negative emotional stimuli [51], the anterior LPP response does vary with stimulus valence such that negative emotions elicit larger amplitudes over the right hemisphere while positive emotions show a similar increase over the left hemisphere [54]. Individual differences in LPP responses have not yet been studied extensively in clinical populations (for a review see [50]), although Zilber and colleagues found larger LLP responses to negative stimuli in adults with greater attachment anxiety [55].

The present study assessed potential PWS genetic subtype differences in brain mechanisms associated with social (faces) versus nonsocial (objects) stimulus processing as well as the ability to distinguish emotional valence (positive vs. negative) of these stimuli as measured by ERPs. As previous data suggest that social deficits in PWS become more pronounced with age [16,56], we focused on adolescents and adults with PWS. We hypothesized that individuals with more typical social functioning would show a larger N170 response to faces than nonsocial stimuli, and that larger differences would be observed in LPP responses to faces with positive versus negative emotions. We also predicted that if people with the two genetic subtypes of PWS subtype differed in their attention to faces versus nonsocial stimuli, such differences should be evident in the amplitude of P3 responses to smiling faces serving as attention targets. The P3 response is not affected by social or emotional content of a stimulus but reflects conscious detection of a less frequent target among more frequent distractors (for a review see [57]). Although exploratory, we also examined subtype differences in LPP responses to positive and negative nonsocial stimuli.

## Method

### Participants

Twenty-four adolescents and young adults with PWS (12 males; mean age = 22.04, standard deviation = 5.60 years) participated in the study. Thirteen participants had the deletion subtype and 11 had the mUPD subtype. Five were left-handed, the rest were right-handed (mean laterality quotient = 0.48, standard deviation = 0.65) as determined by the Edinburgh Handedness Inventory [58]. IQ was assessed by the Kaufman Brief Intelligence Test-2 [59], which was individually administered by trained research assistants. As shown in Table 1, the mean total IQ for the PWS group was 71.04 (standard deviation = 20.91), and although the scores were higher for the mUPD group (mean = 79.91, standard deviation = 27.65) than the deletion group (mean = 63.54, standard deviation = 8.25), the difference failed to reach statistical significance (*P* = 0.08). All participants had normal or corrected-to-normal vision.

**Table 1:** Demographic information for the participant sample

	**Deletion**	**mUPD**	**Total**
*n* (male/female)	13 (7/6)	11 (3/8)	24 (10/14)
Age (years)	22.10 (5.23)	21.96 (6.26)	22.04 (5.60)
Handedness (LQ)	0.47 (0.66)	0.48 (0.66)	0.48 (0.65)
K-BIT IQ	63.54 (8.25)	79.91 (27.65)	71.04 (20.91)
Verbal	72.54 (9.13)	83.27 (21.90)	77.46 (16.79)
Matrices	63.46 (12.95)	80.73 (27.69)	71.38 (22.32)
ADOS (new algorithm)			
Social Affect total	3.31 (3.12)	5.36 (4.95)	4.25 (4.10)
Restricted and Repetitive Behavior total	0.92 (1.38)	1.55 (1.75)	1.21 (1.56)
Social Affect Total + Restricted Repetitive Behavior total	4.23 (3.79)	6.91 (6.41)	5.46 (5.22)
Severity score	2.69 (2.14)	4.00 (3.63)	3.29 (2.93)

ADOS, Autism Diagnostic Observation Schedule; K-BIT, Kaufman Brief Intelligence Test [59]; LQ, laterality quotient [58]; mUPD, maternal uniparental disomy.

Autism-related symptomatology was assessed using Module 3 of the Autism Diagnostic Observation Schedule (ADOS) [60] administered by research-reliable psychologists. The ADOS was scored using the new algorithm that yields separate scores for Social Affect and Restricted, Repetitive Behaviors as well as a total score representing the severity of symptoms [61]. Group differences in ADOS scores failed to reach statistical significance (one-way analysis of variance (ANOVA) *P* = 0.217 to 0.341; see Table 1).

Parents or legal guardians provided written informed consent, and participants with PWS provided written assent. This study was conducted with approval from the Institutional Review Board of Vanderbilt University, in accordance with the Helsinki Declaration of 1975, as revised in 2000 (World Medical Association Declaration of Helsinki 2000).

### Event-related potential task

#### Stimuli

Thirty-two color photographs were included of faces (upright and inverted; from the standardized set by Ekman and Matsumoto [62]) and nonsocial objects (household objects, nonprimate animals). One-half of the social and nonsocial stimuli had positive affective value (for example, a smiling face, a birthday cake), while the other half were negative (for example, an angry face, a mean-looking dog). Each photograph was presented in the center of a computer screen against a black background. From the viewing distance of 90 cm, the stimuli subtended respective visual angles of 8.91° (h) × 6.68° (w).

#### Electrodes

A high-density array of 128 Ag/AgCl electrodes embedded in soft sponges (Geodesic Sensor Net; EGI, Inc., Eugene, OR, USA) was used to record the ERPs. Electrode impedance levels were at or below 40 kΩ as checked before and after testing. During data collection, data were sampled at 250 Hz with the filters set to 0.1 and 30 Hz. All electrodes were referred to vertex and then re-referenced offline during data analysis to an average reference.

### Procedure

The stimuli were presented in an oddball-like paradigm to ensure participants’ continuous attention to the stimuli and their affective content. Photographs from each stimulus category (faces, inverted faces, objects) and emotional content (positive, negative) were presented equally often. Participants were asked to press one button on a hand-held response box in response to smiling faces and another button for all other stimuli (specific button assignment was counterbalanced across the participants). The smiling faces (upright and inverted) appeared on 48 of 144 trials (33%).

Each trial included a 1,000 ms presentation of the stimulus image. The response collection window included up to 2,000 ms from stimulus onset. The intertrial interval was marked by a blank black screen and varied randomly in length between 1,200 and 1,600 ms to prevent habituation and development of trial-onset expectations. Stimulus presentation was controlled by E-prime (v.2.0; PST, Inc., Pittsburgh, PA, USA). The entire task included 144 trials (24 trials × 3 stimulus categories × 2 affective values). On average, the task duration was approximately 10 minutes. A researcher was present in the room to monitor participants’ behavior. During any periods of inattention or motor activity, stimulus presentation was suspended until the participant was ready to continue.

### Data analytic plan

#### Behavioral data

Accuracy and reaction time data were collected for each stimulus condition and submitted to a repeated-measures ANOVA with Subtype (2: deletion, mUPD) × Stimulus Category (3: face, inverted face, nonsocial object) × Emotion (2: positive, negative) factors and Huynh–Feldt correction.

#### Event-related potential

Individual ERPs were derived by segmenting the ongoing electroencephalogram to include a 100-ms prestimulus baseline and an 800-ms post-stimulus interval. Trials contaminated by ocular or movement artifacts were rejected from further analysis using an automated screening algorithm in NetStation (EGI, Inc., Eugene, OR, USA) followed by a manual review. The automated screening criteria were set as follows: for the eye channels, voltage in excess of 140 μV was interpreted as an eye blink and voltage above 55 μV was considered to reflect eye movements. Any electrode with voltage exceeding 200 μV was considered bad. Individual electrodes with poor signal quality were replaced by reconstructing their data using spherical spline interpolation procedures. If more than 15% of the electrodes within a trial were deemed bad, the entire trial was discarded. Trial retention rates were comparable across conditions and groups (mean deletion = 15.85, standard deviation = 3.70; mean mUPD = 17.36, standard deviation = 4.75).

**Figure 1:**
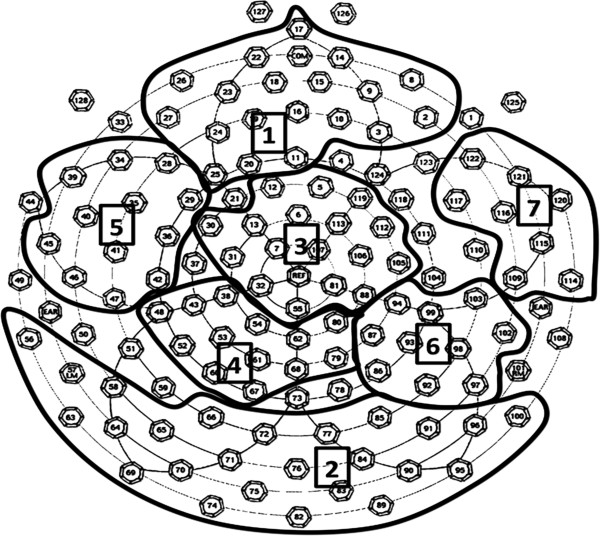
**Electrode map, clusters and corresponding peaks of interest used in the analysis.** 1, frontal cluster (P3); 2, occipito-temporal (N170); 3, central (P3); 4, parietal (P3/late positive potential (LPP)); 5, left fronto-temporal (LPP); 6, right parieto-temporal (LPP); 7, right fronto-temporal (LPP).

Following artifact screening, individual ERPs were averaged and baseline-corrected by subtracting the average microvolt value across the 100-ms prestimulus interval from the post-stimulus segment. To reduce the number of electrodes in the analysis, data from 128 electrodes were submitted to a spatial principle components analysis (PCA) using a covariance matrix and Promax rotation, an objective and replicable statistical approach that identified a small set of virtual electrodes (see Figure 1), each representing a spatially contiguous group of electrodes with similar ERP waveforms (see [63]). Specific electrodes comprising each cluster were identified using the criterion of factor loadings ≥|0.6|.

Clustered data were then submitted to a temporal PCA with Varimax rotation. The temporal PCAs reduced 800 ms (200 time samples) of data to a small set of noncorrelated components accounting for the maximum variance. These components corresponded to the temporal windows of correlated variability in the ERP waveform. The use of the data-driven objective temporal PCA approach reduced the risk of experimenter bias influencing the identification of individual peaks, which is arguably present when visual analysis is used. The number of factors to be used in later analyses was chosen using the Scree Test [64]. Boundaries of individual temporal windows were identified using the criterion of factor loadings ≥|0.6|.

The resulting values were entered into a repeated-measures ANOVA with Subtype (2: deletion, mUPD) × Stimulus Category (3: face, inverted face, nonsocial object) × Emotion (2: positive, negative) × Electrode Cluster (7) factors and Huynh–Feldt correction.

## Results

### Behavioral performance

By analyzing behavioral performance, a main effect of Stimulus was found for accuracy measures, *F*(2,40) = 5.247, *P* = 0.019, partial η^2^ = 0.200. Follow-up pairwise *t* tests indicated that inverted faces were associated with lower response accuracy than objects (79% vs. 86%, *t*(22) = 2.343, *P* = 0.007). For the reaction time, there was an Emotion × Subtype interaction, *F*(1,21) = 4.228, *P* = 0.05, partial η^2^ = 0.168. Follow-up one-way ANOVAs indicated a trend toward longer reaction times to negative stimuli in participants with the mUPD versus deletion subtype (838 ms vs. 706 ms, *P* = 0.066).

### Event-related potential findings

The spatio-temporal PCA identified seven electrode clusters encompassing 105 of 124 electrodes (85%; Figure 1), and five temporal windows accounting for 83.54% of the total variance.

#### Face versus object differences

**Figure 2:**
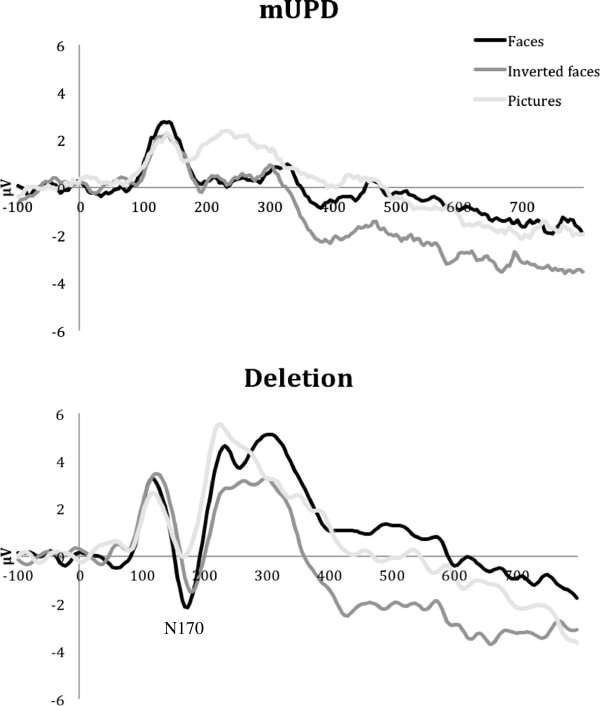
**Occipito-temporal N170 response to faces, inverted faces, and objects by genetic subtype.** mUPD, maternal uniparental disomy.

Analysis of the ERPs in the N170 range (144 to 196 ms) revealed a Stimulus × Electrode × Subtype interaction, *F*(12,252) = 3.133, *P* = 0.006, partial η^2^ = 0.130. Follow-up one-way ANOVA indicated that the two genetic subgroups differed in their amplitudes of the occipito-temporal N170 in response to faces (*F*(1, 22) = 3.648, *P* = 0.042), with larger amplitudes recorded in the deletion group than the mUPD group (Figure 2). Further analyses within each subtype revealed that only participants with the deletion subtype generated larger occipito-temporal N170 responses to faces than objects, *t*(12) = 4.528, *P* = 0.001, *d* = 1.26. The N170 response of the deletion group to inverted faces was smaller than that to faces (*t*(12) = 3.290, *P* = 0.006, *d* = 0.91) and larger than that to objects (*t*(12) = 2.753, *P* = 0.018, *d* = 0.76). No stimulus-related differences reached significance in the mUPD group (*P* = 0.63 to 0.86).

Stimulus differences were also present in the later portion of the waveform (552 to 800 ms) as indicated by a Stimulus × Electrode interaction, *F*(12.252) = 3.423, *P* = 0.030, partial η^2^ = 0.140. Follow-up pairwise *t* tests demonstrated that face stimuli elicited more positive amplitudes than inverted faces at occipito-temporal (*t*(22) = 4.224, *P* < 0.001, *d* = 0.88) and both parietal (*t*(22) = 4.176, *P* < 0.001, *d* = 0.87) and right parieto-temporal (*t*(22) = 3.585, *P* = 0.002, *d* = 0.75) scalp locations, while the reverse direction of differences was observed at the frontal sites (*t*(22) = 5.373, *P* < 0.001, *d* = 1.12). A similar pattern of amplitude differences was observed for faces versus objects (frontal: *t*(22) = 2.597, *P* = 0.016, *d* = 0.53; occipito-temporal: *t*(22) = 2.612, *P* = 0.016, *d* = 0.53; parietal: *t*(22) = 2.366, *P* = 0.027, *d* = 0.48; right parieto-temporal: *t*(22) = 2.221, *P* = 0.036, *d* = 0.45).

#### Emotional valence discrimination

**Figure 3:**
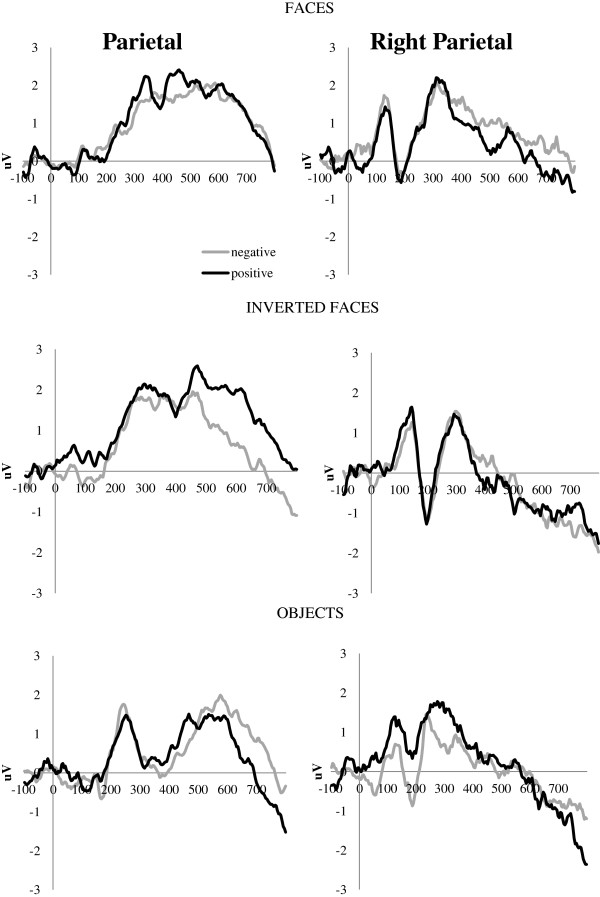
**Posterior late positive potential responses to positive and negative stimuli.** Posterior late positive potential (LPP) responses to positive and negative stimuli (combined sample) at parietal (left column) and right parieto-temporal (right column) scalp locations.

Emotion-related differences in ERP responses were observed in the 344 to 592 ms window corresponding to the early LPP response in the form of a Stimulus × Emotion × Electrode interaction, *F*(12,252) = 2.338, *P* = 0.047, partial η^2^ = 0.100. A larger parietal LPP was present in response to positive than negative objects, *t*(23) = 2.235, *P* = 0.035, *d* = 0.456 (Figure 3). There were no significant differences in the amplitudes between positive and negative upright or inverted faces. At the right parieto-temporal cluster, inverted faces elicited smaller LPP amplitudes than upright faces (positive: *t*(23) = 2.968, *P* = 0.007, *d* = 0.619; negative: *t*(23) = 2.435, *P* = 0.023, *d* = 0.508) or objects (positive: *t*(23) = 2.706, *P* = 0.013, *d* = 0.564; negative: *t*(23) = 1.989, *P* = 0.059, *d* = 0.415). However, there were no significant differences between positive versus negative emotional valence for any of the three stimulus categories.

**Figure 4:**
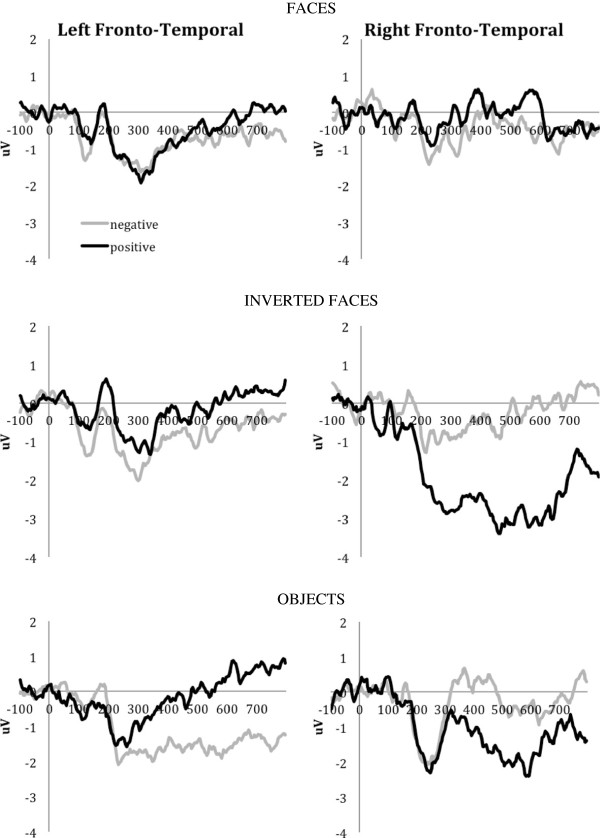
Left and right fronto-temporal late positive potential for the combined sample.

Examination of the anterior LPP response at the left and right fronto-temporal clusters revealed more positive amplitudes in response to positive versus negative objects on the left (*t*(23) = 2.044, *P* = 0.053, *d* = 0.417), while the direction of differences was reversed at the right hemiscalp sites, *t*(23) = 3.389, *P* = 0.003, *d* = 0.692 (Figure 4). Similarly, larger right frontal LPP responses were observed for negative than positive inverted faces, *t*(23) = 2.803, *P* = 0.010, *d* = 0.584. No emotion-related differences reached significance for upright faces.

#### Smile detection (attention to stimuli)

**Figure 5:**
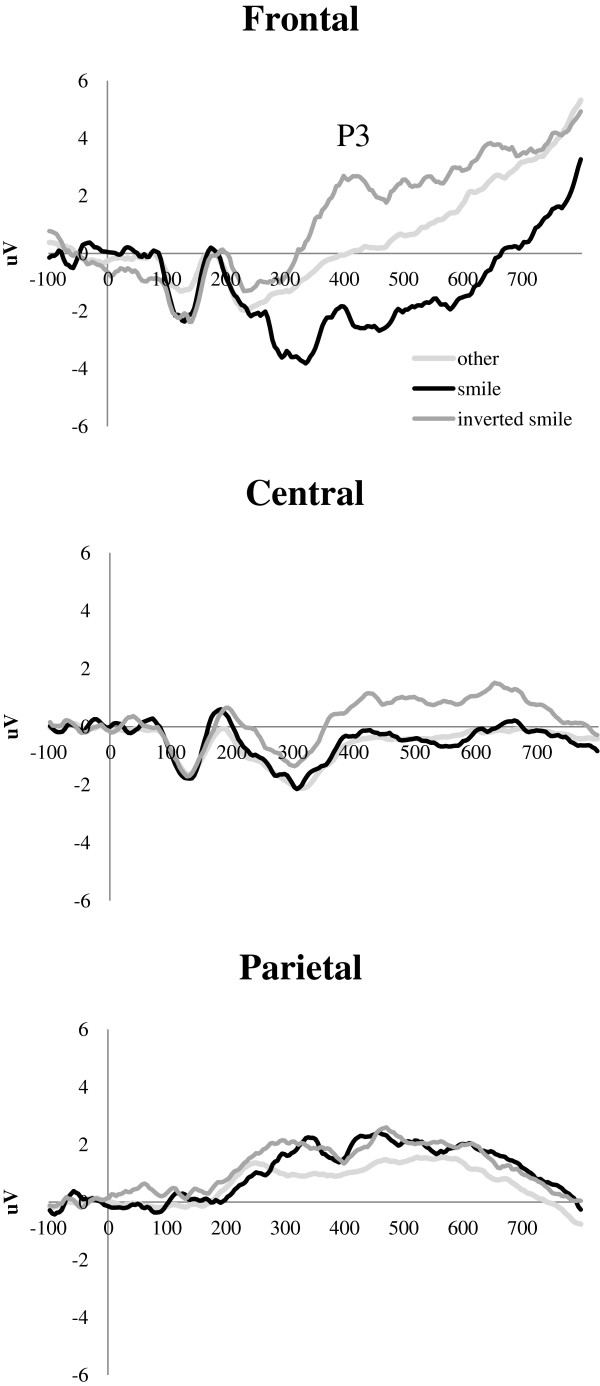
P3 responses at frontal, central, and parietal locations for the combined sample.

To evaluate potential differences in attention to the stimuli, a separate *post-hoc* analysis of the Stimulus × Emotion × Electrode interaction in the 344 to 592 ms window focused on the combined response to smiling upright and inverted faces (targets) versus other stimuli at frontal, central, and parietal electrode clusters. Pairwise *t*-tests with Bonferroni correction indicated that the expected increase in positivity associated with the target/oddball stimulus detection was observed only at frontal and central but not parietal locations (Figure 5). A direct comparison of amplitudes between the two target stimulus types indicated larger responses for inverted than upright smiles (frontal: *t*(22) = 4.479, *P* < 0.0001, *d* = 0.934; central: *t*(22) = 4.703, *P* < 0.0001, *d* = 0.981). There was no significant increase in P3 amplitude for the upright smiling faces compared with other stimuli.

### Brain–behavior connections

To explore whether autism symptomatology in PWS is related to neural responses differentiating faces from objects or positive from negative emotional content, we correlated posterior N170, and parietal and fronto-temporal LPP measures with the ADOS severity score.

Greater severity scores were associated with smaller (less negative) N170 responses to inverted smiling faces (*r* = 0.414, *P* = 0.050) and larger right parieto-temporal LPP responses to upright (*r* = 0.406, *P* = 0.049) and inverted (*r* = 0.445, *P* = 0.033) negative faces. Higher ADOS severity scores were also related to larger right fronto-temporal LPP responses (*r* = 0.410, *P* = 0.047) and smaller left fronto-temporal LPP responses (*r* = 0.425, *P* = 0.039) to upright negative faces.

## Discussion

Although people with PWS are at increased risk for autism symptomatology, especially those with mUPD subtype, most prior work in this area has focused on restricted and repetitive behaviors. In contrast, this study probed social and emotional processing across the two major genetic subtypes of PWS with methods and stimuli previously used to examine social processing in persons with ASD. In doing so, we provide the first direct evidence of genetic subtypes differences in the social perception of individuals with PWS.

The experimental design provided an opportunity to assess both basic face perceptual processes (face vs. object, upright vs. inverted face) as well as attention to the stimuli (smiling faces vs. all other stimuli) and their emotional (positive vs. negative) content without placing excessive cognitive demands on participants. At the behavioral level, participants with the deletion or mUPD subtypes of PWS did not differ in their ability to perform the task of detecting smiling faces among negative faces and nonsocial objects with positive and negative valence, as evidenced in their similar behavioral accuracy and reaction time. Similarly, Halit and colleagues also found no subtype differences in behavioral performance on face processing tasks [43]. Although the mUPD group in our study did show slower responses to negative stimuli, this nonsignificant trend needs further study. All participants were more successful in separating faces from nonsocial objects, but less accurate in identifying smiling faces among other facial expressions. This pattern of findings suggests that even though individuals with PWS are similar to typical populations in terms of treating faces as a separate perceptual category, they also resemble individuals with ASD in their difficulty with evaluation of facial expressions.

Although the two groups did not differ on behavioral indices, psychophysiological measures revealed important genetic subtypes differences in neural processes involved in the perceptual analyses of social and nonsocial images. In contrast to those with mUPD subtype, only those with deletions demonstrated the expected larger posterior N170 responses to faces than objects. ERPs of individuals with the deletion subtype thus resembled those elicited by faces and objects in the typical population, while the lack of face-object differences in ERPs of participants with the mUPD subtype is more consistent with findings in ASD.

Individuals with the deletion subtype also differentiated between upright and inverted faces; however, instead of the expected enhancement of the N170 to inverted faces, that response was reduced, falling between objects and faces. Similar direction of amplitude differences for upright versus inverted faces in persons with the deletion subtype was observed by Halit and colleagues [43], but in that study it did not reach significance, probably due to a smaller sample size (*n* = 8 vs. *n* = 13 in the present study). Conversely, there were no orientation-related differences in ERPs to faces in persons with the mUPD subtype. This finding is inconsistent with the results from Halit and colleagues [43] and could be attributed to sample size differences as well as to the greater variety of the facial stimuli in the present study (16 vs. 3 facial identities, inclusion of male as well as female faces) and the use of faces with emotional rather than neutral expression.

The absence of face inversion-related enhancement in our sample of persons with PWS (both the deletion and mUPD subtype) is consistent with prior findings reported in ASD groups [42,65]. These similarities in brain responses are further strengthened by the observed correlation between ERPs and ADOS severity scores where larger (that is, more typical) N170 responses to inverted smiling faces were associated with reduced autism symptomatology.

Alternatively, genetic subtype differences in N170 response could be attributed to the different gender composition of these groups. Indeed, the mUPD group was predominantly female, while the deletion group was more balanced in gender, but included slightly more males. However, no gender differences have been reported in accuracy of face recognition [66], and in our study there were no differences in accuracy of behavioral responses to faces between the two subtype groups. Prior studies of gender differences in N170 responses only report effects for the topographic distribution of N170, with females showing more bilateral activation than males, who elicit right-lateralized N170 [67,68]. Further, the posterior electrode cluster used for N170 analyses was selected using objective data-driven procedures that identified patterns of brain activity common to the entire study sample, which included a comparable number of males and females. The resulting cluster was nearly symmetrical in shape and included homologous occipito-temporal locations from both hemispheres (with the exception of three additional temporal electrodes in the left hemisphere), and therefore would be unlikely to bias the outcomes toward a specific gender. Finally, even if the electrode cluster was biased toward a specific gender, its bilateral distribution should have increased the likelihood of larger N170 responses being observed in the predominantly female mUPD group, yet plotted data and statistical effects indicate the opposite. We therefore conclude that observed group differences in N170 response are due to genetic subtype and not the gender composition of the samples.

In contrast to the N170 responses, no genetic subtype differences emerged in indices of emotion discrimination. All participants showed differential brain responses to positive and negative stimuli in the LPP range. Parietal LPP differences associated with emotional content were present for objects only (larger for positive stimuli) and observed mainly at midline and left-hemisphere scalp locations, suggesting that participants with both genetic subtypes of PWS were more sensitive to the arousal level and emotional content of nonsocial objects than faces. For the right parieto-temporal cluster, a larger LPP was observed for faces and objects than inverted faces, but no emotion-related differences were present.

These findings are in line with previous studies suggesting that posterior LPP responses may reflect the arousal value of the stimuli rather than the specific emotional valence. LPP amplitudes would thus be larger for more arousing than neutral stimuli but would not necessarily vary based on positive or negative emotions [51,53]. Among the three stimulus types, inverted faces elicited the least amount of arousal in participants, consistent with their low ecological significance. At the same time, nonsocial objects may be associated with greater arousal and more varied emotional response, as evidenced by the modulation of the parietal LPP. This pattern of results is consistent with recent reports suggesting that emotional faces may not be the most effective stimuli for eliciting affective reactions [53]. Nevertheless, individual differences in right parieto-temporal LPP responses to negative upright and inverted faces were related to autism symptomatology in participants with PWS, where higher ADOS severity scores were associated with larger LPPs.

In contrast to the parietal LPP, anterior LPP responses were expected to differentiate between emotional content, especially because the experimental task required explicit evaluation of the emotional content [54]. This expectation was supported by the results of the present study: participants with PWS demonstrated hemisphere-specific emotion discrimination. Incidentally, our electrode clusters (identified using a data-drive spatial PCA approach) overlapped those defined *a priori* as optimal for the frontal LPP [54]. For nonsocial objects, larger anterior LPP amplitudes were found for negative versus positive stimuli at right fronto-temporal locations, while at left fronto-temporal sites the LPP amplitude was enhanced for positive versus negative items. Inverted faces also showed the right-hemisphere bias for negative emotional content, as reflected by increased right fronto-temporal LPP amplitudes for negative versus positive expressions. However, processing of the upright faces remained unaffected by emotional content, despite the explicit task instructions to attend to that stimulus dimension. Yet brain–behavior correlations indicated that greater autism symptomatology was associated with larger right anterior and smaller left anterior LPP responses elicited by the negative upright faces.

The lack of significant differences in neural responses indicating emotion discrimination in upright faces could be attributed to insufficient attention to the stimuli. The relatively high behavioral accuracy of responses to the smiling faces suggests that participants both understood and performed the task. However, analyses of the attention-specific P3 response revealed the expected enhanced amplitude to smiling faces (targets) only at frontal and central sites but not parietal sites. This scalp distribution of stimulus condition differences corresponds to the anterior P3a, thought to reflect involuntary orienting to rare stimuli [57]. Smiling faces might thus have attracted attention due to their overall lower probability in the stimulus stream. The absence of condition differences for the parietal P3 response could be attributed to insufficient task engagement and reduced voluntary attention to the stimuli. Furthermore, the anterior P3a was more pronounced for the inverted than upright smiling faces. It is possible that inverted faces attracted more processing resources due to their unusual physical appearance, and thus their emotional content was also processed to a greater extent. Conversely, the upright faces received only minimal attention that did not involve explicit emotion identification.

Although our study generated novel findings that replicate and extend previously reported results, the present study has several limitations. It is possible that our findings are sample specific because the sample size was relatively small for each subtype, and the groups were not matched on gender. The mUPD group also included individuals with a wider range of intellectual functioning than the deletion group. While including IQ as a covariate in the statistical analyses did not alter the outcomes related to group differences in the N170 response to faces, further studies are needed with larger numbers that are more homogeneous in intellectual functioning and gender. An additional possible concern is that the social and nonsocial stimuli varied greatly in their arousal value and emotional valence. While the faces were selected from a standardized set [62], the nonsocial stimuli were not obtained from a set (for example [69]) and were instead selected based on salient features of the PWS phenotype. Given their intellectual disabilities, for example, we selected items that had a simple figure-ground organization and were appropriate for the participants’ developmental level. Given their hyperphagia, we avoided food stimuli and instead used stimuli with emotional content tailored to the known likes and dislikes of the study sample (for example, cute vs. menacing animals). Even so, diverse nonsocial stimulus types have been used in prior studies of face vs. object processing (for example, toys, cars, butterflies, and so forth), and our findings of face–object discrimination in the deletion group but not in the mUPD group argue against the possibility that our nonsocial stimuli were insufficient in creating a contrast between N170 responses to faces and objects. Similarly, emotion-related differences were found primarily for the nonsocial stimuli, not in standardized upright emotional faces, suggesting that our nonstandardized stimuli were sufficient to elicit an affective reaction. Finally, we only used a single measure of ASD features (ADOS severity score) rather than a wider range of tests targeting social skills. This choice was motivated by the need to balance reasonable statistical power with the available sample size. We therefore selected the gold-standard measure of autism symptomatology. However, future studies with larger samples are needed to more specifically delineate the relationship between brain responses to emotional faces versus objects and social functioning in persons with various genetic subtypes of PWS.

## Conclusions

This study provides the first neural evidence of genetic subtype differences in the social perceptions of individuals with PWS. Those with mUPD, but not deletions, generated brain responses that resembled those of persons with ASD in their lack of a face-specific increase in the amplitude of the posterior N170, suggesting potential alterations in social perceptual processes that may contribute to increased ASD symptomatology in this group. This finding holds promise for future research that connects patterns of gene expression in mUPD to functional outcomes in social perception. Importantly, the two PWS genetic subtypes do not appear to differ in their processing of emotional content. All participants with PWS demonstrated more extensive processing of emotional valence in nonsocial than face stimuli, and for the latter they exhibited a potential bias toward negative social affect. Although further work is needed, these results suggest possible mechanisms that underlie social difficulties in persons with PWS, and also offer potential new treatment targets or outcome measures for future trials aimed at ameliorating negative mood or social dysfunction.

## Abbreviations

ADOS: Autism Diagnostic Observation Schedule; ANOVA: Analysis of variance; ASD: Autism spectrum disorders; ERP: Event-related potential; IQ: Intellectual quotient; LPP: Late positive potential; mUPD: Maternal uniparental disomy; PWS: Prader-Willi syndrome; PCA: Principal components analysis.

## Competing interests

The authors declare that they have no competing interests.

## Authors’ contributions

APK designed the experimental paradigm, performed statistical analyses, interpreted data, and drafted the manuscript. DJ carried out the electroencephalography data collection, coordinated ERP data processing, and provided data quality control. EMD conceived of the study, participated in its design and interpretation, and helped to draft the manuscript. All authors read and approved the final manuscript.
